# A case report of mixed-phenotype acute leukemia with atypical *BCR::ABL1 e13a3* fusion gene

**DOI:** 10.1097/MD.0000000000046329

**Published:** 2026-05-12

**Authors:** Yan Zhou, Mei Liu, Yunlu Zhao, Rui Zhang, Qi Guo, Zhanlong Wang, Yanbo Nie, Jingci Yang, Min Shi

**Affiliations:** a Department of Clinical Laboratory, The Second Hospital of Hebei Medical University, Shijiazhuang, P.R. China; b Department of Cardiovascular Disease, Shinshu University School of Medicine, Matsumoto, Japan; c SINO-US Diagnostics Lab, Tianjin Enterprise Key Laboratory of AI-aided Hematopathology Diagnosis, Tianjin, China; d Hebei Key Laboratory of Laboratory Medicine, Shijiazhuang, P.R. China.

**Keywords:** atypical transcript, *BCR::ABL1* fusion, mixed-phenotype acute leukemia, Philadelphia chromosome

## Abstract

**Rationale:**

Mixed-phenotype acute leukemia (MPAL) is a type of acute leukemia which is characterized by immunophenotypic features of myeloid, T-lymphoid, and/or B-lymphoid differentiation. The Philadelphia chromosome-positive (Ph+) MPAL, a rare subtype of MPAL, represents <1% of adult acute leukemia cases and typically has a poor prognosis. Here we report a very unique case of MPAL with Ph + atypical *e13a3 breakpoint cluster region (BCR)::ABL1* fusion protein and provide new perspectives on the diagnosis and management of Ph + MPAL.

**Patient concerns:**

We present a 64-year-old male who experienced high-grade fever, nasal congestion, and a runny nose 20 days after contracting a cold. A chest computed tomography revealed double pneumonia. The patient was presented with an anemic appearance, scattered bleeding spots, and ecchymoses, with no superficial lymph nodes palpable.

**Diagnoses:**

The patient was diagnosed as MPAL with atypical *e13a3 BCR::ABL1* transcripts by morphology, flow cytometry, cytogenetic and molecular biology analyses.

**Interventions:**

The patient was treated with a combination therapy of VCD (Bortezomib, Cyclophosphamide, Dexamethasone), Venetoclax, and Dasatinib, supplemented with liver protection, immune modulation, and supportive care.

**Outcomes:**

He achieved remission after 2 lines of therapy but subsequently experienced a sudden relapse 3 months later.

**Lessons:**

At present, there is no established consensus on the treatment of Ph + MPAL, and reports on cases with the atypical *e13a3 BCR::ABL1* fusion are particularly scarce. This finding will bring new insights and references for the diagnosis and treatment of Ph + MPAL.

## 1. Introduction

Mixed-phenotype acute leukemia (MPAL) is a rare subgroup of acute leukemia, accounts for < 0.5% of all cases of acute leukemia.^[1]^ According to the 2022 WHO classification, MPAL is divided into 5 subtypes: MPAL with *BCR::ABL1* rearrangement, MPAL with rearranged *KMT2A*, MPAL with B-cell/myeloid features not otherwise specified, MPAL with T-cell/myeloid features not otherwise specified, and acute undifferentiated leukemia. MPAL often has a poor prognosis and difficulty in both diagnostic and therapeutic clinicals.^[2]^ It refers to leukemia cells expressing immunophenotypic features of at least 2 cell lineages-defining markers of myeloid, T-lymphoid, and/or B-lymphoid.^[3,4]^

The Ph + is generated when the proto-oncogene abelson murine leukemia 1 (*ABL1*) gene translocates to the breakpoint cluster gene (*BCR*).^[5]^ The translocation t(9;22)(q34;q11) results in the formation of a fusion gene called *BCR::ABL1*.^[6]^ However, the presence of Ph + has been found to be a factor associated with a worse prognosis.^[7,8]^ Owing to the nonspecific marker and pathological characteristics of leukemia cells, diagnosis is confronted with more challenges for atypical Ph + MPAL cases. Additionally, there is still no consensus on how to treat it optimally.

Here, we report a case of 64-year-old male patient with atypical Ph + MPAL. From our knowledge, this is the first report of the Ph + MPAL case with atypical *e13a3 BCR::ABL1* transcripts.

## 2. Materials and methods

Materials and methods used in the study are available in Supplement Data S1, Supplemental Digital Content, https://links.lww.com/MD/Q817. All samples received written informed consent from the ethical committee of the Second Hospital of Hebei Medical University (ethics number: 2024-R547).

## 3. Results

### 3.1. General examination results of the case

The patient, a 64-year-old male, developed a fever reaching up to 38°C, nasal congestion, and a runny nose 20 days after contracting a cold, leading to a diagnosis of double pneumonia on chest computed tomography. He was subsequently admitted to the hematology department of our hospital. His medical history includes hypertension for the past 6 years and a left frontoparietal hemangioma resection 3 years ago.

Physical examination revealed a body temperature of 36.8°C, a pulse rate of 84 beats per minute, a respiratory rate of 21 breaths per minute, and a blood pressure of 140/89 mm Hg. The patient was presented with an anemic appearance, scattered bleeding spots, and ecchymoses, with no superficial lymph nodes palpable. He exhibited sternum tenderness and coarse breath sounds in both lungs. The abdomen was flat and soft, with no palpable liver or spleen under the ribs, and no swelling in the lower limbs.

**Figure 1.**
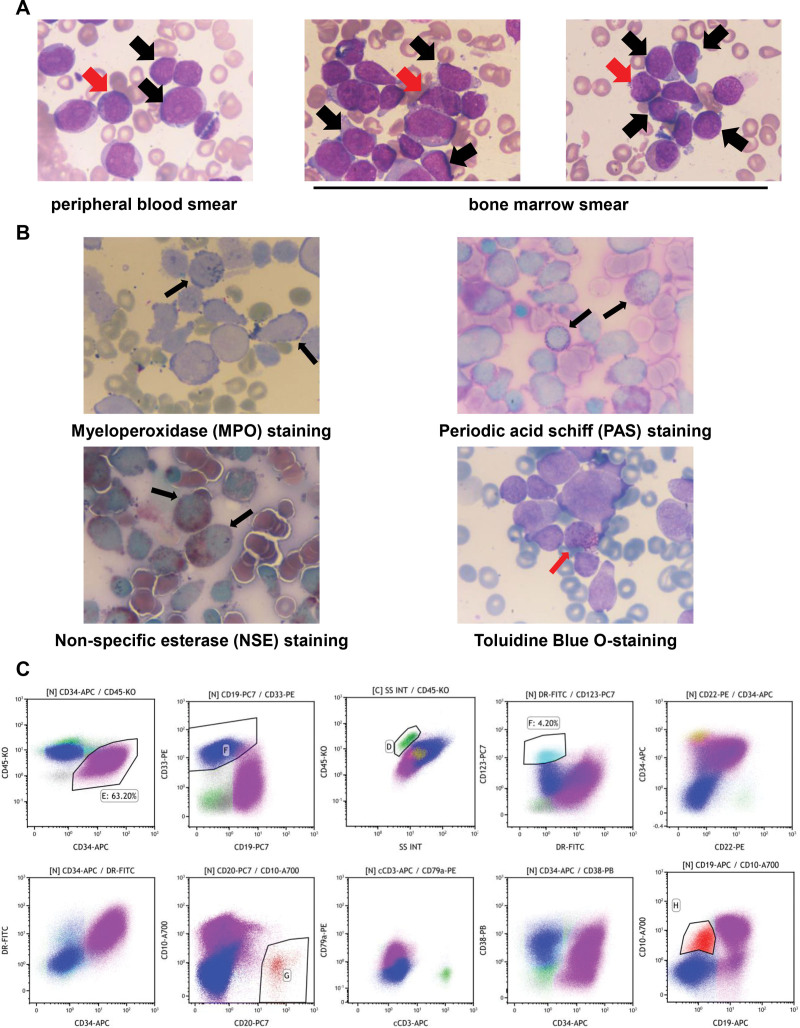
Pathological changes and flow cytometry analysis results of the case. (A) Photography of Wright-Giemsa staining for peripheral blood smear or bone marrow smear. Black arrows indicate blast cells, and red arrows indicate basophilic precursor cells. (1000×). (B) Photography of multiple cytochemical staining for marrow smear. (1000×). MPO staining and NSE staining showing positive in part of blast cells (black arrow); PAS staining showing positive in another part of blast cells (black arrow). Toluidine blue staining shows positive in basophil precursor cells (red arrow). (C) Flow cytometry analysis results. Purple marked cells are abnormal early B lymphocytes; blue marked cells are abnormal myeloid cells; green marked cells’ classification is not determined; cyan cells are basophils. MPO = myeloperoxidase, NSE = nonspecific esterase, PAS = periodic acid-schiff.

A full blood count showed a red blood cell count of 2.23 × 10^12^/L, hemoglobin of 79 g/L, platelet count of 18 × 10^9^/L and white blood cell count of 189.3 × 10^9^/L (54% blasts and 13% basophil precursor cells). Microscopy examination of a peripheral blood smear with Wright − Giemsa staining revealed a large number of blast cells without granules in cytoplasm (Fig 1A, black arrow) and some basophil precursor cells with cytoplasm containing variable blackish purple granules (Fig 1A, red arrow). A bone marrow aspirate and biopsy were performed for further evaluation before therapy. Blood chemistry revealed lactate dehydrogenase levels of 3121 U/L, alanine aminotransferase at 110.2 U/L, and aspartate transaminase at 153.5 U/L.

### 3.2. Bone marrow aspirate, biopsy and flow cytometry analyses

A bone marrow aspirate revealed hypercellularity with 81% blast cells (Fig 1A, black arrow), divided into 2 distinct groups. The first group consisted of small cells with round nuclei, dense chromatin, variably visible nucleoli, and sparse, blue-staining cytoplasm. The second group comprised larger cells with oval or irregular, sometimes twisted and folded nuclei, fine chromatin, 1 to 2 nucleoli, moderately abundant, blue-staining cytoplasm, and occasional purple-red granules. Basophil precursor cells made up 9% of the sample (Fig 1A, red arrow). Only 2 megakaryocytes were observed, and there was a significant decrease in platelets..

Bone marrow biopsy analysis Bone marrow biopsy analysis confirmed hypercellularity with over 90% proliferation of blast cells, and reticular fiber staining was graded MF-2, focal, consistent with the pathological characteristics of leukemia.

Cytochemical stains revealed that a portion of the blast cells tested positive for myeloperoxidase staining (Fig 1B, black arrow), while others showed positive reactions to periodic acid-schiff staining and nonspecific esterase staining (Fig 1B, black arrow). A few basophil precursor cells also tested positive for Toluidine Blue staining (Fig 1B, red arrow).

Flow cytometric analysis (Fig 1C, Supplement Data S2, Supplemental Digital Content, https://links.lww.com/MD/Q817) identified that 95% of the cells analyzed were abnormal, categorized into 3 distinct groups. The first group consisted of abnormal early B lymphocytes (purple), comprising 63.2% of the cells. Immunophenotyping showed CD34 (+), CD123 (+), CD38 (+), HLA-DR (+), CD10 (+), CD22 (+), CD19 (+), CD20 (-) and CD79a (+) with a small amount of CD33 expression. The second group, making up 27.6% of the cells, consisted of abnormal myeloid cells (blue). Immunophenotyping showed CD34 (−), CD123 (+), CD38 (+), HLA-DR (−), CD64 (+), CD11c (+), CD14^dim^, CD33 (+), CD15 (+), CD11b (+) and CD13 (+). The third group, which accounted for 11.3% of the cells, included abnormal early cells with a phenotype of CD34 (+), CD22 (-), CD19 (+), CD33 (+), but the cell lineage was undetermined (green). T-lymphoid markers were CD7 (−), CD5 (−), CD2(−), CD3 (−). Additionally, basophils, representing about 4.2% of the cells (cyan) existed. Based on these analyses, a diagnosis of MPAL (B/myeloid) was confirmed.

### 3.3. Cytogenetic and molecular biology analyses

**Figure 2.**
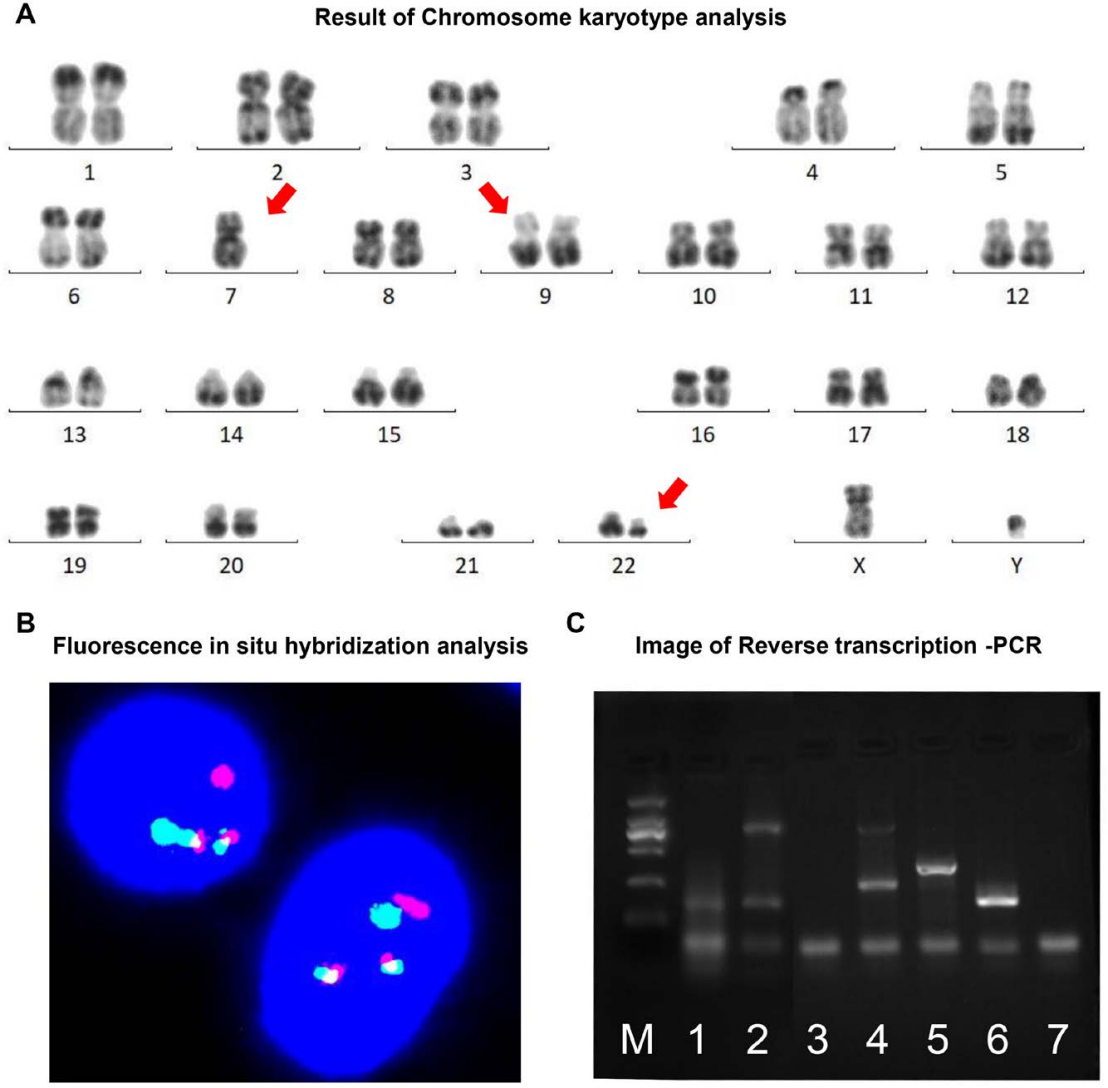
Chromosome and gene analyses of the case. (A) Result of chromosome karyotype analysis. (B) Image of fluoMPrescence in situ hybridization analysis. Red: ABL1; green: BCR. (C) Imaging of reverse transcription-PCR products after electrophoresis. M: marker, 1000 bp Marker; 1: The patient’s BCR::ABL1 e13a3 PCR product; 2. Positive control of e13a3; 3: Negative control; 4: P210 positive control; 5: P190 positive control; 6: P230 positive control; 7: Negative control. BCR = breakpoint cluster region.

Chromosome karyotype analysis revealed a complex metaphase karyotype of 45, XY, −7, t(9;22) (q34.1;q11.2) in 17 cells and a normal karyotype of 46, XY in 3 cells (Fig. 2A). Fluorescence in situ hybridization testing (FISH) detected a BCR (green) and ABL1 (red) fusion signal in 94% of the cells (n = 200), significantly exceeding the established threshold of 2.54% (Fig. 2B). Gene mutation screening identified copy number variation in the *IKZF1* and *CUX1* genes, heterozygous mutation of p.R1048X and p.Q1228Rfs*33 in the *BCORL1* genes (Supplement Data S3, Supplemental Digital Content, https://links.lww.com/MD/Q818). RT-PCR confirmed an atypical *BCR::ABL1 e13a3* PCR product of 169bp (Fig. 2C). Based on these findings, the diagnosis was established as MPAL with atypical *e13a3 BCR::ABL1* transcripts.

### 3.4. Treatment and intervention process

After the diagnosis of MPAL with atypical *e13a3 BCR::ABL1* transcripts, the patient received a combination therapy of VCD (bortezomib, cyclophosphamide, dexamethasone), venetoclax, and dasatinib to treat the Ph + MPAL. This treatment was supplemented with liver protection, immune modulation, and supportive care. One month later, a bone marrow biopsy indicated a significant reduction in primitive cell counts to 0.5%. Flow cytometry revealed that abnormal early B lymphocytes comprised approximately 0.19% of total cells. The myeloid primitive cells accounted for about 0.78%, showing no significant phenotypic abnormalities. FISH analysis indicated that the *BCR::ABL* fusion gene was below the 2.54% threshold, and RT-PCR showed a *BCR::ABL* fusion gene/internal reference gene quantitative ratio of 12.49%. The primary disease was considered resolved, allowing the patient to be discharged.

Thirty-seven days later, the patient was readmitted for consolidation therapy using the same treatment regimen, augmented with antiemetics and liver protection. A subsequent bone marrow examination again showed a low primitive cell count (0.5%). Flow cytometry indicated that CD34 and CD117 positive cells made up approximately 0.022% of nucleated cells, with no significant abnormalities in the tested phenotypes and no grouping of abnormal B lymphocytes. RT-PCR revealed the *BCR::ABL* fusion gene (e13a3)/internal reference gene quantification ratio was 0.19%.

Two months later, the patient was readmitted with left lower limb knee pain and a sensation of fullness in the right lower limb. A bone marrow puncture revealed highly active proliferation of marrow nuclear cells and a large presence of primitive cells. Flow cytometric analysis showed that abnormal primitive early B lymphocytes constituted about 88% of the bone marrow nucleated cells, while no abnormal myeloid primitive cells were detected. RT-PCR demonstrated a *BCR-ABL* (e13a3 type)/internal reference gene ratio of 232.62% (*BCR-ABL*: 2,382,349 copies/ *ABL*: 1,024,147 copies), confirming a relapse of MPAL. The family members of the patient opted to discontinue further treatment and requested his discharge from the hospital.

## 4. Discussion

According to the fifth edition of the WHO diagnostic criteria, MPAL is categorized into 2 types: biphenotypic and bilineage.^[1]^ Biphenotypic MPAL is characterized by a single population of abnormal progenitors (morphological blasts) expressing antigens from 2 or more lineages. In contrast, bilineage MPAL involves 2 distinct populations of abnormal progenitors, each from a different lineage.^[3]^ For a diagnosis of biphenotypic MPAL, both lineage cells must meet the specific diagnostic criteria, and the combined percentage of the 2 lineages of blast cells must be ≥ 20% of the nuclear cells without a minimum threshold for a single lineage. Additionally, conditions such as acute myeloid leukemia (AML) with *RUNX1::RUNX1T1* fusion, AML with *CBFB::MYH11* fusion, myeloid/lymphoid neoplasms with eosinophilia and specific gene rearrangements, and the blast phase of chronic myeloid leukemia (CML) must be excluded from the diagnosis.

In this case, morphological analysis of the bone marrow aspirate identified 2 distinct lineages of progenitor cells: 1 myeloid and the other lymphoid, with a notably high proportion of basophilic precursor cells. Flow cytometry analysis showed that these lineages of blast cells distinctly expressed B-lineage and myeloid markers. Additionally, 6.8% of the cells were identified as basophils. These findings align with the characteristics of biphenotypic MPAL. Further genetic and molecular biology analyses revealed a chromosomal karyotype with anomalies including deletion 7 and translocation t(9;22) (q34.1;q11.2), along with the detection of an atypical *e13a3 BCR::ABL1 f*usion protein. Based on these results, the diagnosis was confirmed as MPAL with t(9;22) (q34.1;q11.2) *BCR::ABL1* (atypical e13a3).

This disease is exceptionally rare, making it vital to distinguish it from AML and the blast phase of CML. Interestingly, some AML cases with the t(8;21) translocation can exhibit multiple B-cell markers, yet these cases are not classified as biphenotypic due to the presence of only 1 lineage’s markers, thus ruling out a diagnosis of AML.^[9]^ It is also significant that this patient displayed a noticeable proportion of basophils in both peripheral blood and bone marrow smears. The presence of the BCR::ABL1 fusion gene resulting from the translocation t(9;22)(q34;q11.2) is typically associated with the blast phase of CML.^[10,11]^ Moreover, when the patient underwent a resection for a left frontoparietal hemangioma 3 years ago, the blood routine showed no abnormalities, and the patient had no history of CML nor exhibited splenomegaly. Therefore, the blast phase of CML can be conclusively excluded.

MPAL with *BCR::ABL1* fusion accounts for < 0.5% of all cases of acute leukaemia and 15% to 20% of all cases of MPAL, making *BCR::ABL1* fusion the most common cytogenetic abnormality in MPAL.^[1]^ Most transcripts have resulted from chromosomal breakpoints in breakpoint cluster region (BCR) introns 1, 13, or 14 and ABL1 intron 1 (known as e1a2, e13a2, or e14a2, respectively). Depending on the specific breakpoints, different *BCR::ABL1* fusion proteins such as p210, p190, p230, and atypical forms can be generated, with the *BCR::ABL1* p190 protein more commonly found in MPAL patients than the p210 variant.^[12]^ There is extraordinarily little literature related to the cytogenetic and molecular abnormalities of MPAL with atypical *BCR::ABL1*.^[13–15]^ Rare *BCR::ABL1* transcripts, accounting for <1% of CML cases, e19a2 (39.8%), e13a3/e14a3 (20.5%), and e1a2 (16.9%) are the 3 most frequent transcript types.^[16]^ Liu et al reported a 62‐year‐old male with Ph^+^ (atypical e13a2 *BCR‐ABL1* fusion protein) MPAL, which presented with recurrent and intense bone pain due to bone marrow necrosis.^[15]^ The atypical *BCR::ABL1 e13a3* fusion in our case has not been reported yet. The e13a3 transcript is generated through the direct fusion of exon e13 (b2) from the *BCR* gene with exon a3 from the *ABL1* gene, leading to the exclusion of exon a2 in the ABL1 gene.^[17]^ This exon encodes the SH3 domain, which is essential for the negative regulation of *BCR::ABL1* kinase activity, suggesting that the e13a3 transcript may modify *BCR::ABL1* mediated signaling pathways.^[18]^ The absence of the SH3 domain may impact downstream signaling, including the *STAT5* pathway, thereby affecting the proliferative characteristics of leukemic cells.^[19]^ Current research suggests that this transcript may influence the response to tyrosine kinase inhibitors, molecular remission rates, and treatment-free remission following discontinuation of therapy.^[20]^

Few studies have documented the presence of complex karyotypes in conjunction with atypical *BCR::ABL1* transcripts.^[16]^ Ray et al^[21]^ were the first to report a case of MPAL where monosomy 7 and the *BCR::ABL1* gene fusion coexisted, highlighting the translocation t(9;22)(q34;q11.2) as an indicator of poor prognosis. Similarly, in our case, the coexistence of t(9;22)(q34;q11.2) and monosomy 7 along with atypical BCR::ABL1 suggests a potential for poor prognosis.

At present, there is no established consensus on the treatment of Philadelphia chromosome-positive Ph + MPAL, and reports on cases with the atypical *e13a3 BCR::ABL1* fusion are particularly scarce. For MPAL with *BCR::ABL1,* treatment often involves tyrosine kinase inhibitors combined with ALL chemotherapy regimens, followed by allogeneic stem cell transplantation when possible.^[17]^ Studies indicate that MPAL patients with the Ph + chromosome generally have a poor prognosis, with a median survival time of 8 months, compared to 139 months for those with normal karyotypes. Ph + MPAL exhibits characteristics of both acute lymphoblastic leukemia (ALL) and AML. Therefore, the leukemia cells may not be fully sensitive to treatment regimens targeting ALL or AML alone. The combination of vincristine + cyclophosphamide + dexamethasone is an important chemotherapy regimen for the treatment of ALL, mainly used to consolidate and enhance treatment and salvage treatment. Venetoclax has shown significant efficacy in both AML and ALL, while dasatinib (inhibiting *BCR:: ABL1*) and vinaclotide (inhibiting BCL-2) have a strong synergistic effect, which may effectively “starve” and “kill” cancer cells. In this case, the paient was treated with VCD supplemented by hepatoprotection, immune modulation, and supportive therapy. Here, the VCD (vincristine + cyclophosphamide + dexamethasone) regimen is a crucial chemotherapy combination for treating MPAL, primarily used for consolidation and intensification therapy as well as salvage therapy. Despite initial remission following treatment with the VCD + Ven + dasatinib regimen, the patient ultimately relapsed. The origin of blast cells in MPAL from primitive hematopoietic stem cells, which exhibit lineage variation, makes chemotherapy alone insufficient for disease eradication. Consequently, allogeneic stem cell transplantation is recommended in most cases.^[22]^

Current systematic investigations into the genomic and transcriptomic characteristics of Ph + MPAL remain limited.^[23]^ In this case, the presence of mutations in *IKZF1, CUX1*, and *BCORL1* with each bearing definite or potential clinical significance, suggests a poor prognosis. *IKZF1* encodes a zinc finger-leacht plays a role in chromatin remodeling and is essential for the development of all lymphoid cell lineages.^[24]^ The presence of *IKZF1* deletions has been associated with an increased relapse rate in B-cell precursor ALL (BCP-ALL).^[25]^ Yan et.al performed targeted sequencing of 18 genes in 31 cases of MPALBCR::ABL1 and found frequent *IKZF1* mutations in MPAL*BCR::ABL1.*^[26]^ Additionally, The *CUX1* gene is an important tumor suppressor in AML and myelodysplastic syndrome, and its inactivation is a key event in the occurrence and development of leukemia, research reported that CUX1 deficiency is associated with poorer survival rates in myeloid tumors.^[27]^ Lastly, Study has shown that *BCORL1* gene truncation mutation can lead to gene product inactivation, which is related to the pathogenesis of AML.^[28]^ In this case, our patient achieved remission after 2 lines of therapy but subsequently experienced a sudden relapse. It may be related to the coexistence of variations in the *IKZF1, CUX1*, and *BCORL1* genes. Which is worth further research.

In conclusion, the precise diagnosis and classification of MPAL are extremely important. Comprehensive analysis using various methods such as morphology, immunophenotype, genetics, and molecular biology is essential to facilitate precise clinical treatment and prognosis assessment. The presence of the atypical e13a3 *BCR::ABL1* transcript in this MPAL patient is extremely rare. This discovery offers new perspectives on the diagnosis and management of Ph + MPAL.

## Author contributions

**Conceptualization**: Min Shi.

**Investigation**: Yan Zhou, Mei Liu, Rui Zhang, Qi Guo, Zhanlong Wang, Yanbo Nie, Jingci Yang.

**Methodology**: Min Shi.

**Resources**: Min Shi.

**Writing – original draft**: Yan Zhou, Mei Liu, Yunlu Zhao.

**Writing – review & editing**: Min Shi, Yunlu Zhao.

## Supplementary Material
