# Supplementary material for: A case report of mixed-phenotype acute leukemia with atypical BCR::ABL1 e13a3 fusion gene
Source: Medicine (Baltimore). 2026 May 12;104(49):e46329. doi: 10.1097/MD.0000000000046329 (PMC12689148; doi:10.1097/MD.0000000000046329)
Supplement: Supplementary file 1 [file medi-104-e46329-s001.docx]

**Supplement Data S1: Materials and Methods**

***2.1 Morphology analysis***

Bone marrow smears were stained using Wright-Giemsa, allowing for the observation of cell morphology and differential counting under a microscope. Additional morphological analyses were conducted using Myeloperoxidase (MPO) staining, Periodic Acid Schiff (PAS) staining, Non-Specific Esterase (NSE) staining, and Toluidine Blue O staining. The bone marrow biopsy underwent routine processing with formalin fixation and was stained with hematoxylin and eosin.

***2.2 Flow cytometric analysis***

Cell suspensions were prepared and stained for flow cytometry analysis within 24 hours after collecting the bone marrow aspiration samples. For the analysis, 50-mL aliquots of the cell suspensions were treated with fluorescently labeled monoclonal antibodies, including myeloid markers of MPO, CD13, CD33, CD64, CD14, CD15, CD117, CD11b and CD11C, B-lymphoid markers of CD79a, CD10, CD19, CD20 and cCD22, T-lymphoid markers of CD7, CD5, CD2 and CD3, Non-lineage-specific markers of CD34, CD38, CD123 and HLA-DR(Becton Dickinson [BD] Biosciences, San Jose, CA, USA). The cells were incubated with these antibodies for 30 min before 2.5 mL of ammonium chloride was added for 10 min to lyse the red blood cells. Subsequently, the cells were centrifuged, and the resulting cell pellet was washed with phosphate-buffered saline containing 0.1% bovine serum albumin and 0.1% sodium azide. The final cell pellet was resuspended in 0.5 mL of phosphate-buffered saline containing 1% formaldehyde (electron microscope grade, Polysciences, Warrington, PA, USA). The cells were then analyzed using a FACSCalibur flow cytometer (BD Biosciences). Data from this analysis were processed using CellQuest software (BD Biosciences).

***2.3 Cytogenetic analysis***

The chromosome specimens were obtained from bone marrow samples according to conventional methods following 24- and 48-hour cultures at 37˚C. The images of chromosome karyotype were collected on a ZEISS imager Z2 (ZEISS, Oberkochen, Germany) and then analyzed with MetaClient software (ZEISS). When possible, a minimum of 20 metaphases were examined. The R-banding chromosome karyotypes were documented according to the 2013 International System of Human Cytogenetic Nomenclature (ISCN 2013). Abnormal clones were identified by the presence of at least two cells with the same structural abnormalities or extra chromosomes, or by three or more cells showing the loss of the same chromosome.

***2.4 Fluorescence in situ hybridization testing***

The probes used for fluorescence in situ hybridization (FISH) testing, specifically designed to detect BCR:: ABL1 fusion gene, with the ABL1 gene covering 9q34.11-13, approximately 600kb, and the BCR gene covering 22q11.22-23, totaling 1000kb upstream and downstream. The probes are manufactured by Anping Pharmaceutical Technology Co., Ltd (Guangzhou, China). In these tests, the ABL and BCR genes are labeled red and green respectively, with their fusion appearing yellow.

***2.5 Reverse transcription-PCR and sequencing***

The total RNA of mononuclear cells in the specimens was extracted and reverse transcribed into cDNA. The gene was then amplified using a PCR machine, and the resulting product was analyzed via electrophoresis. After the purification, the amplified product was sequenced using the ABI BigDye V3.1 kit on an ABI 3730XL sequencer. The sensitivity of this sequencing method for this study is set at a detection limit of 10%. The sequence of primers is:

BCR-F: AGAACATCCGGGAGCAGCAGAAGAA

ABL-R: CCATTGTGATTATAGCCTAAGACCCGGAG.

**Supplement Data S2: The results of flow cytometric analysis**

| **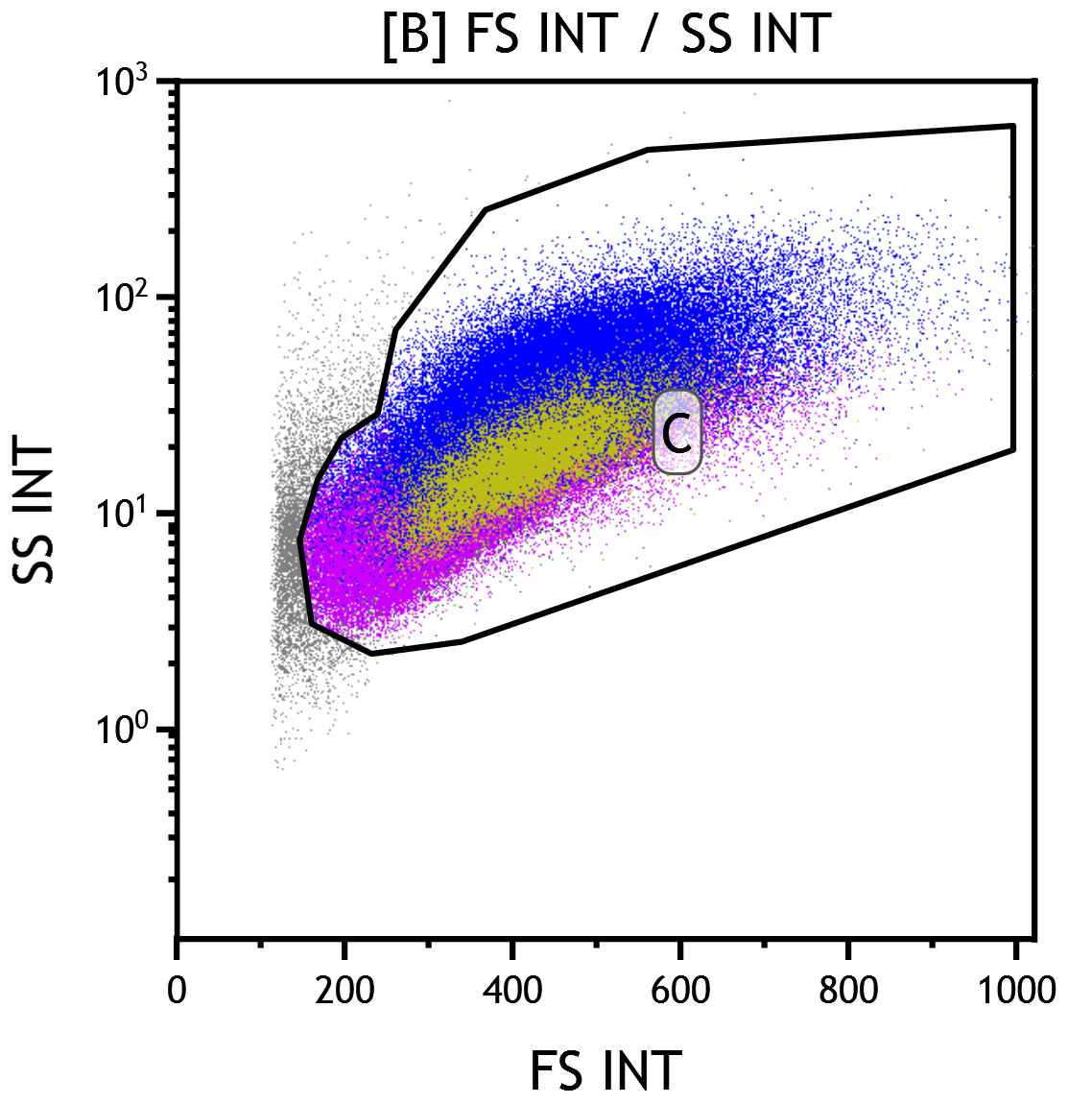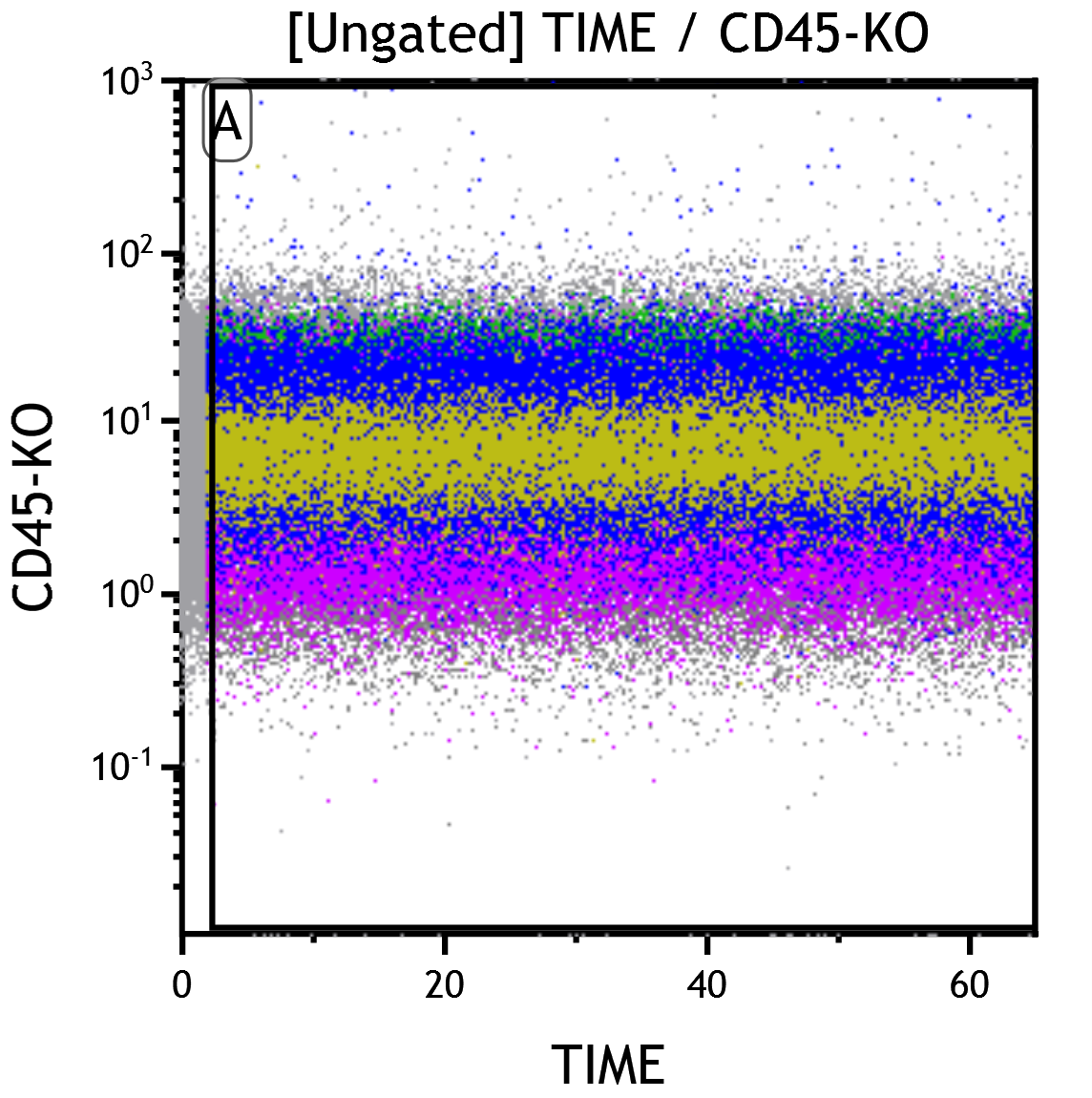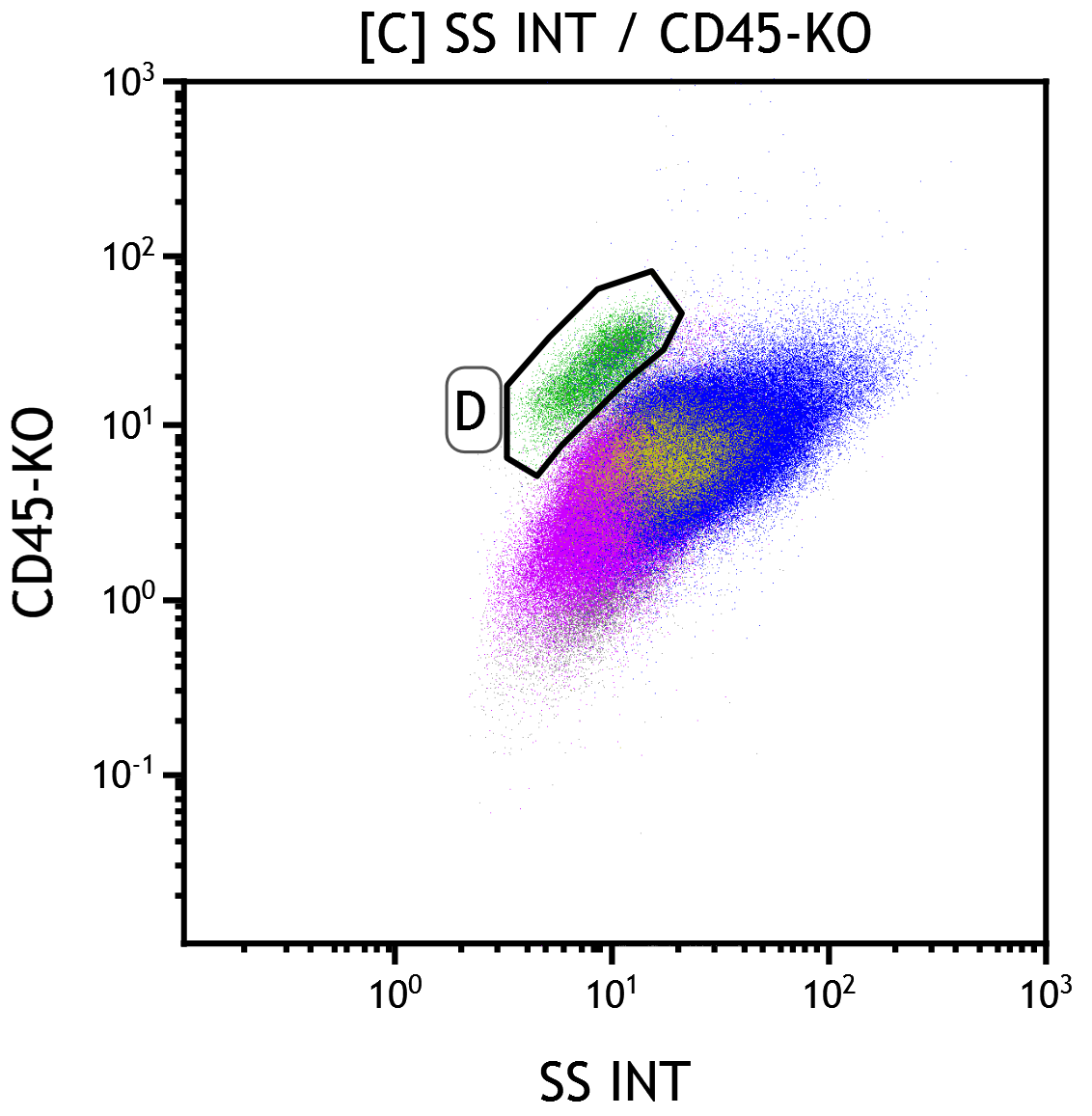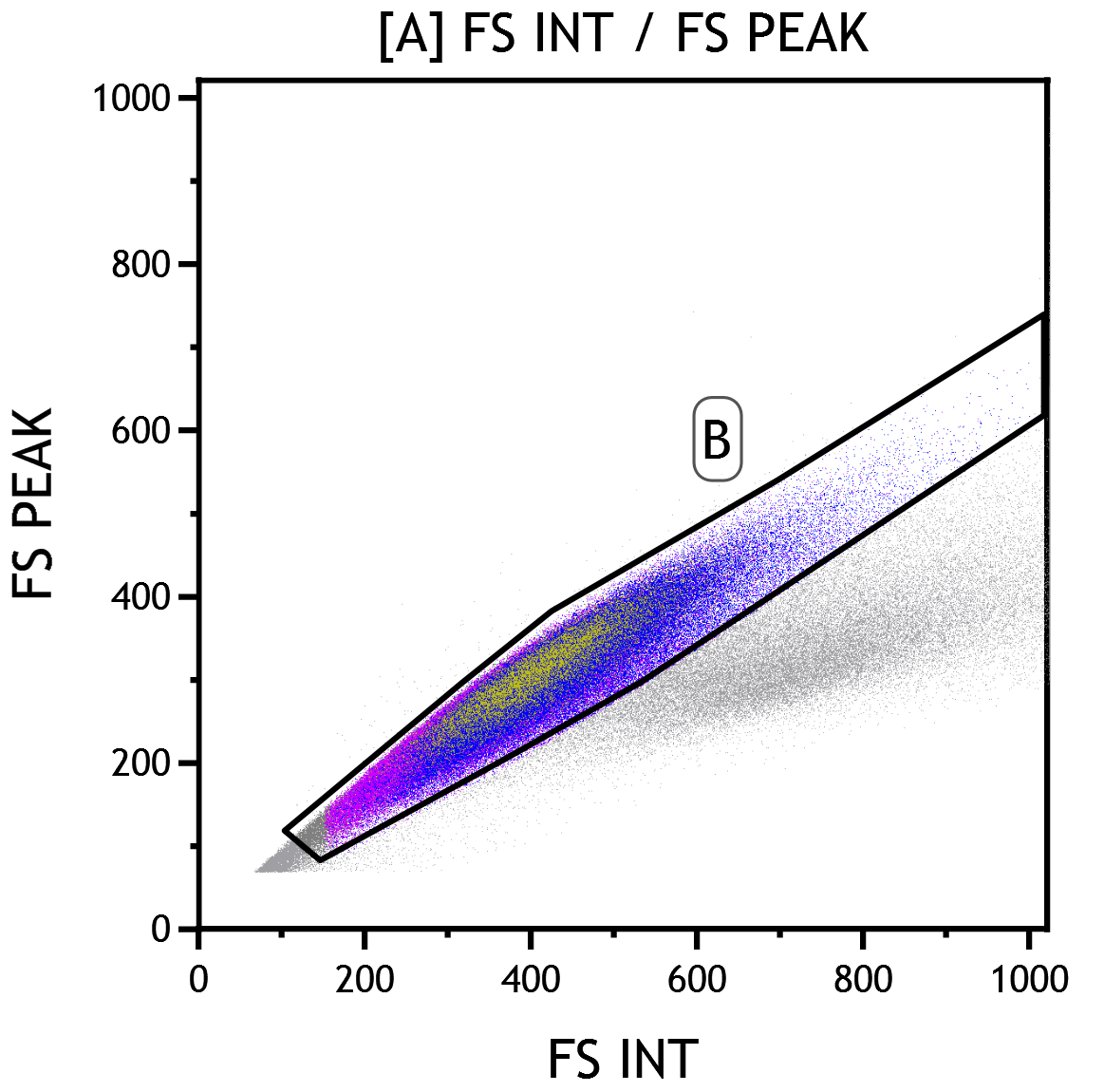**  **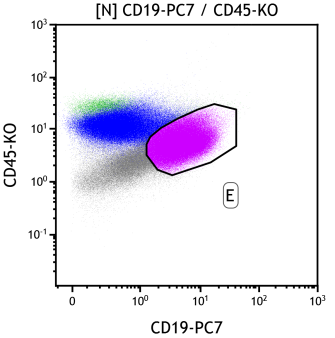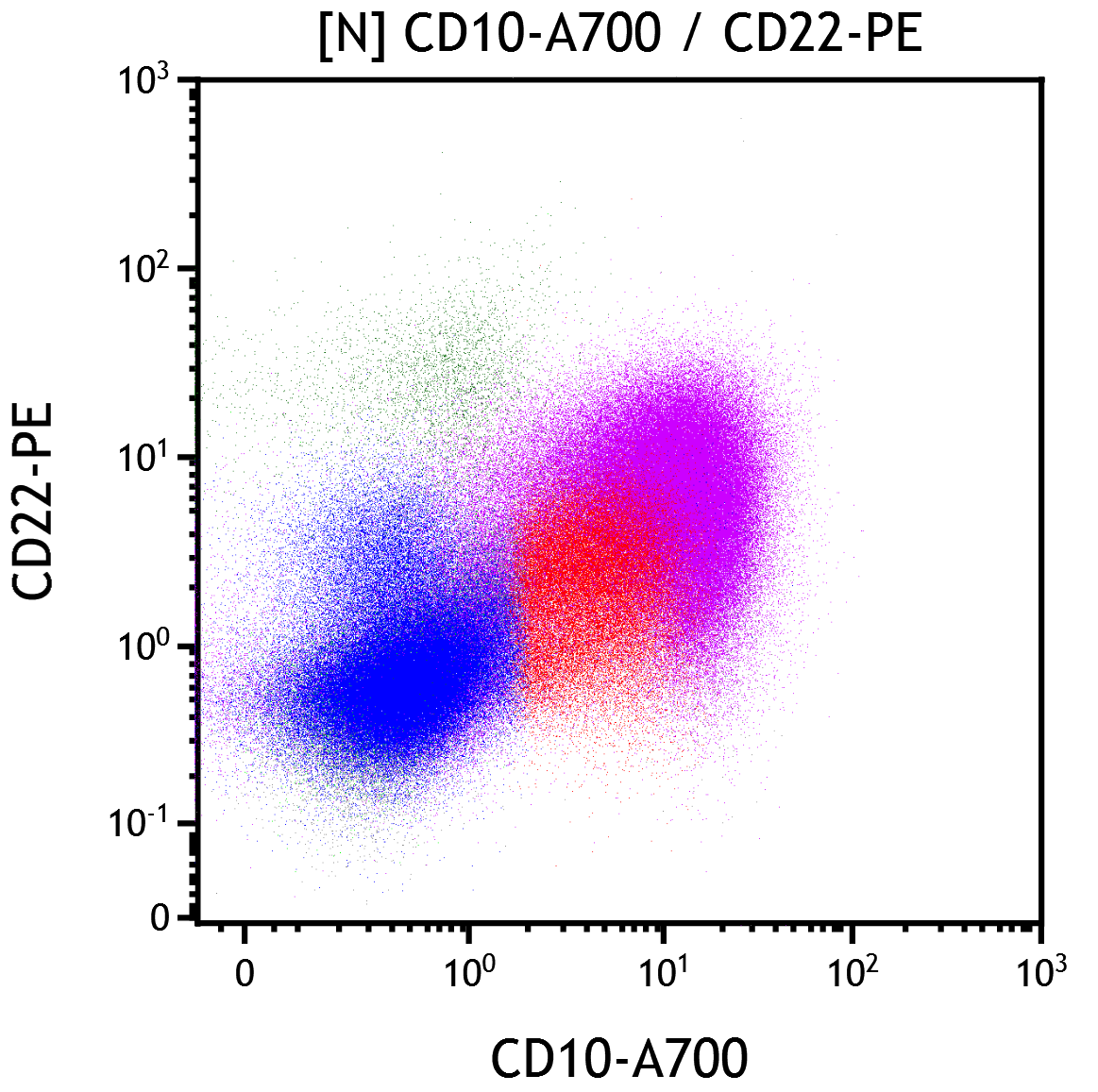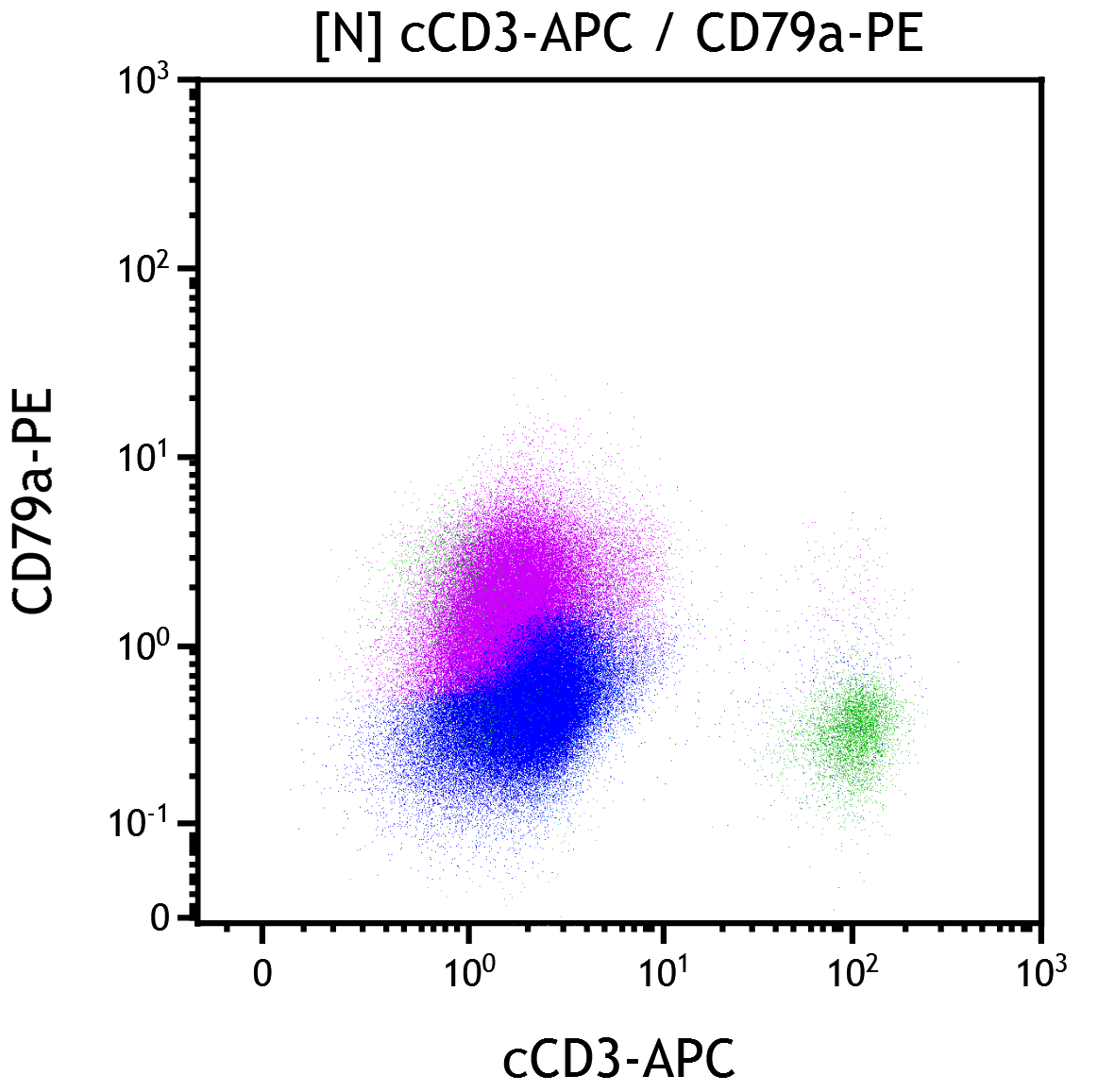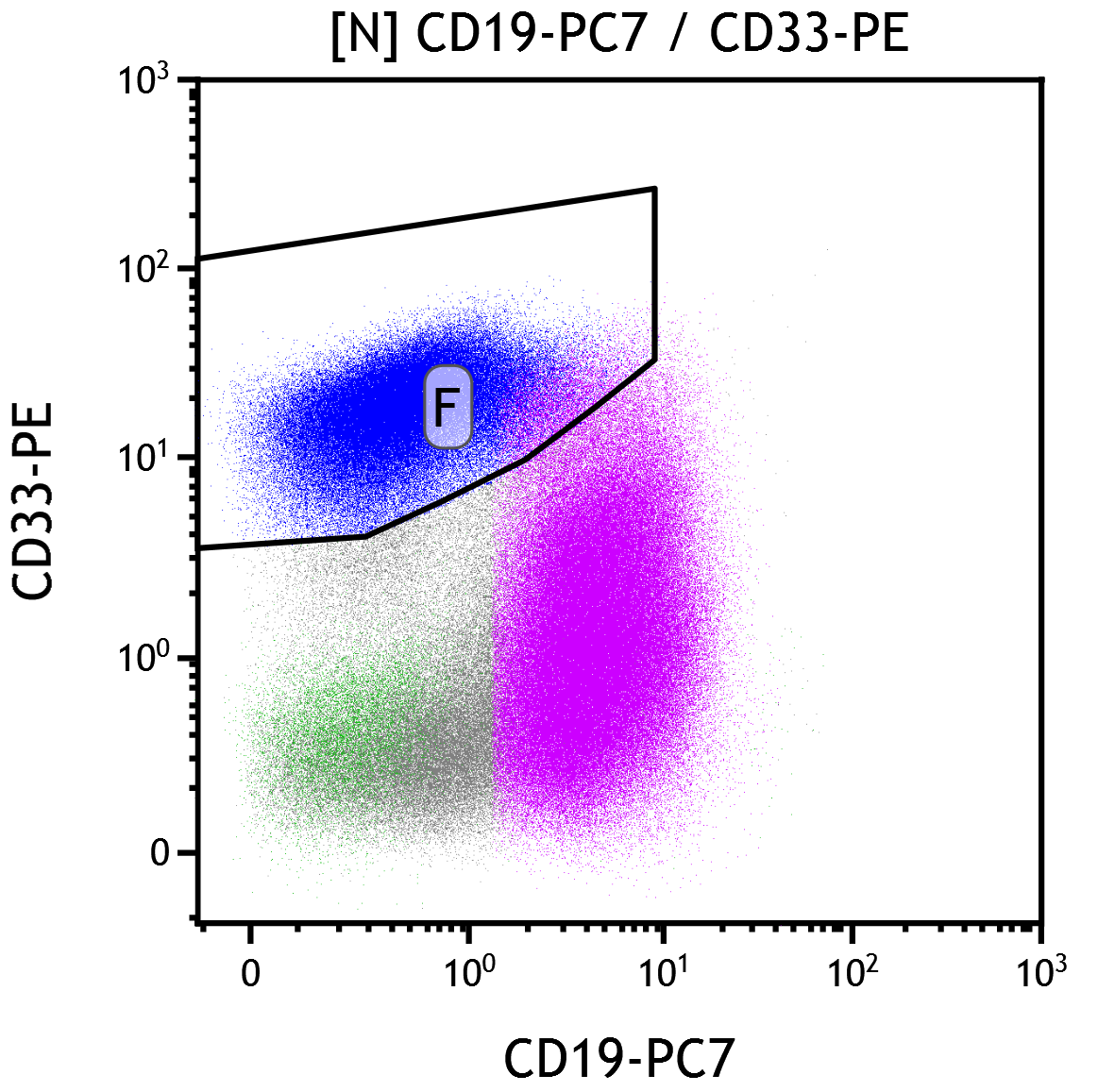**  **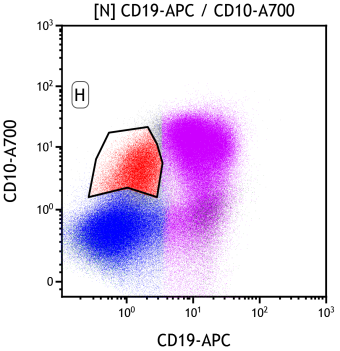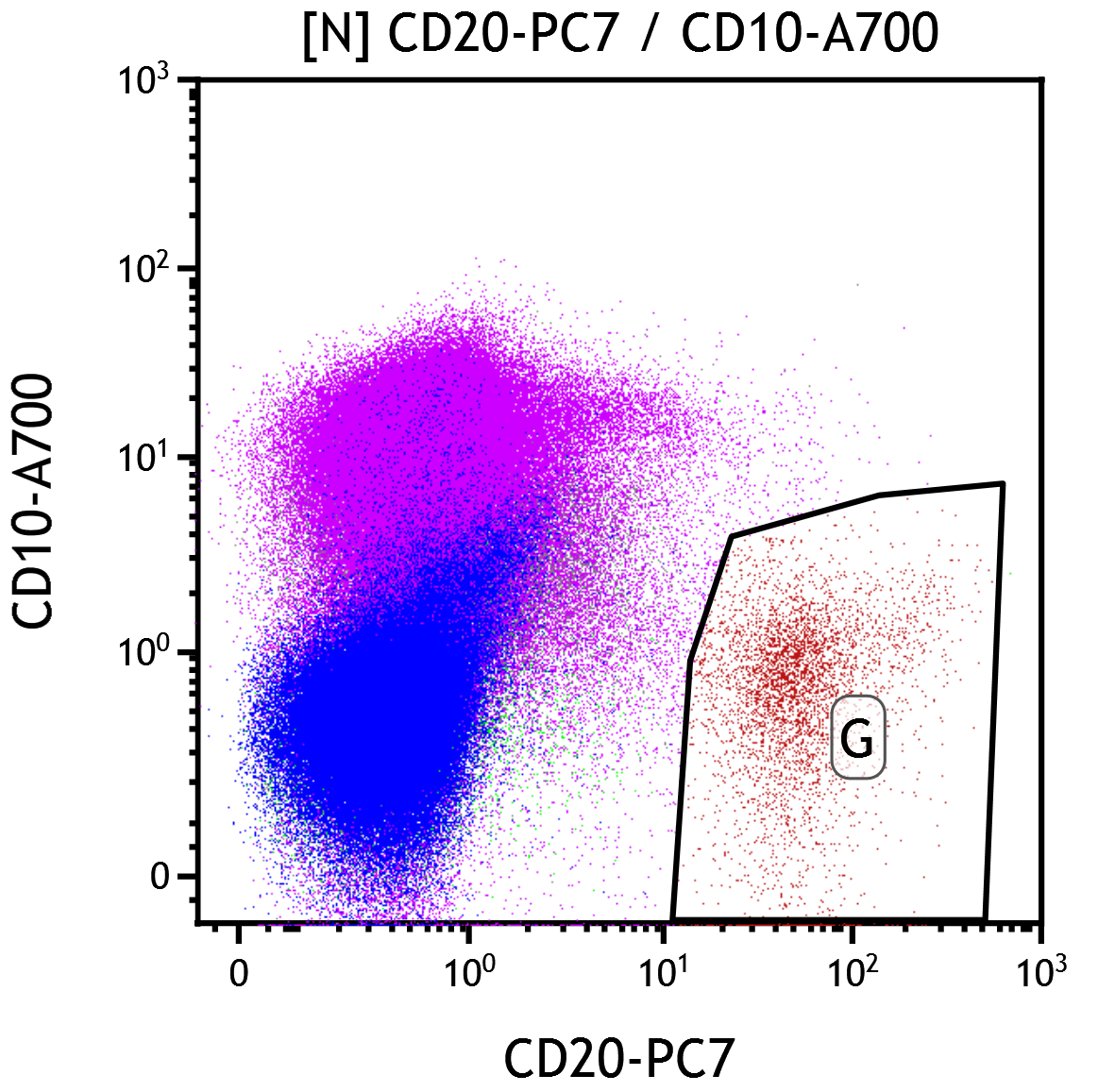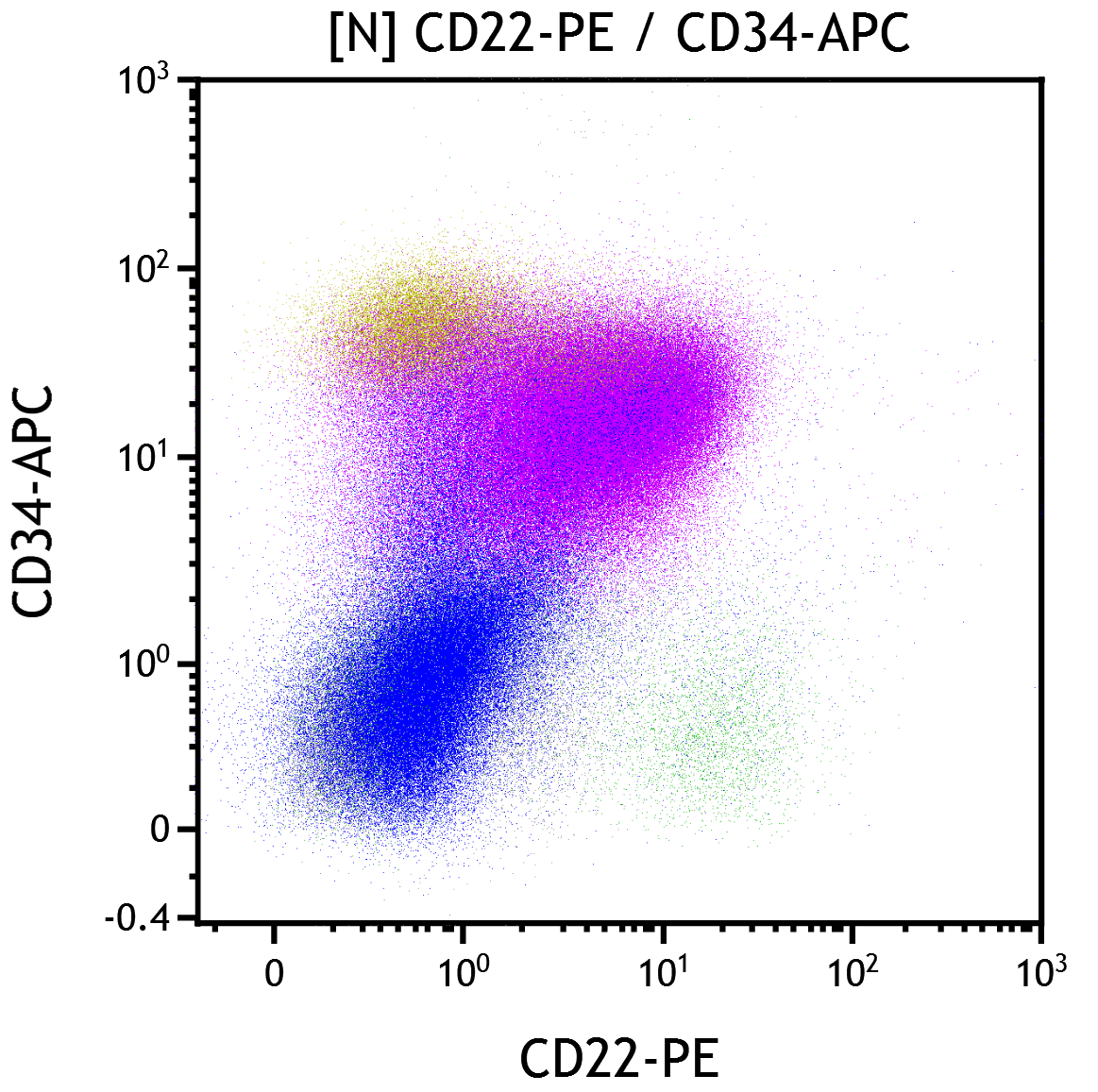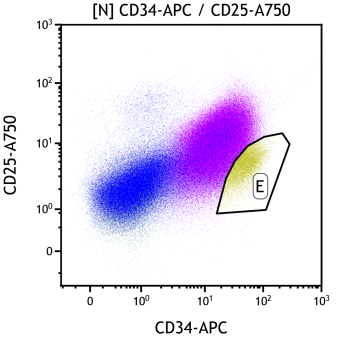**  **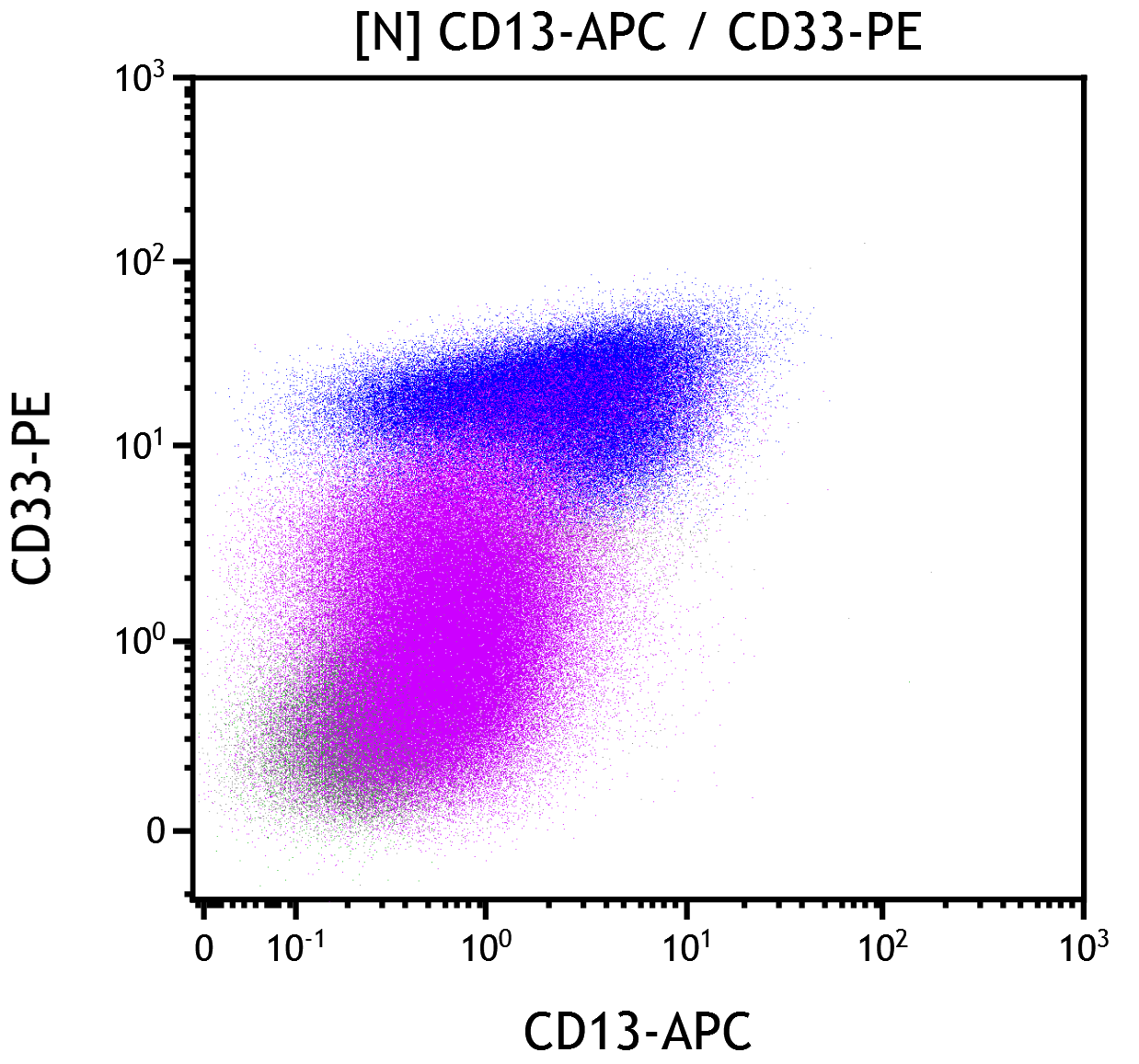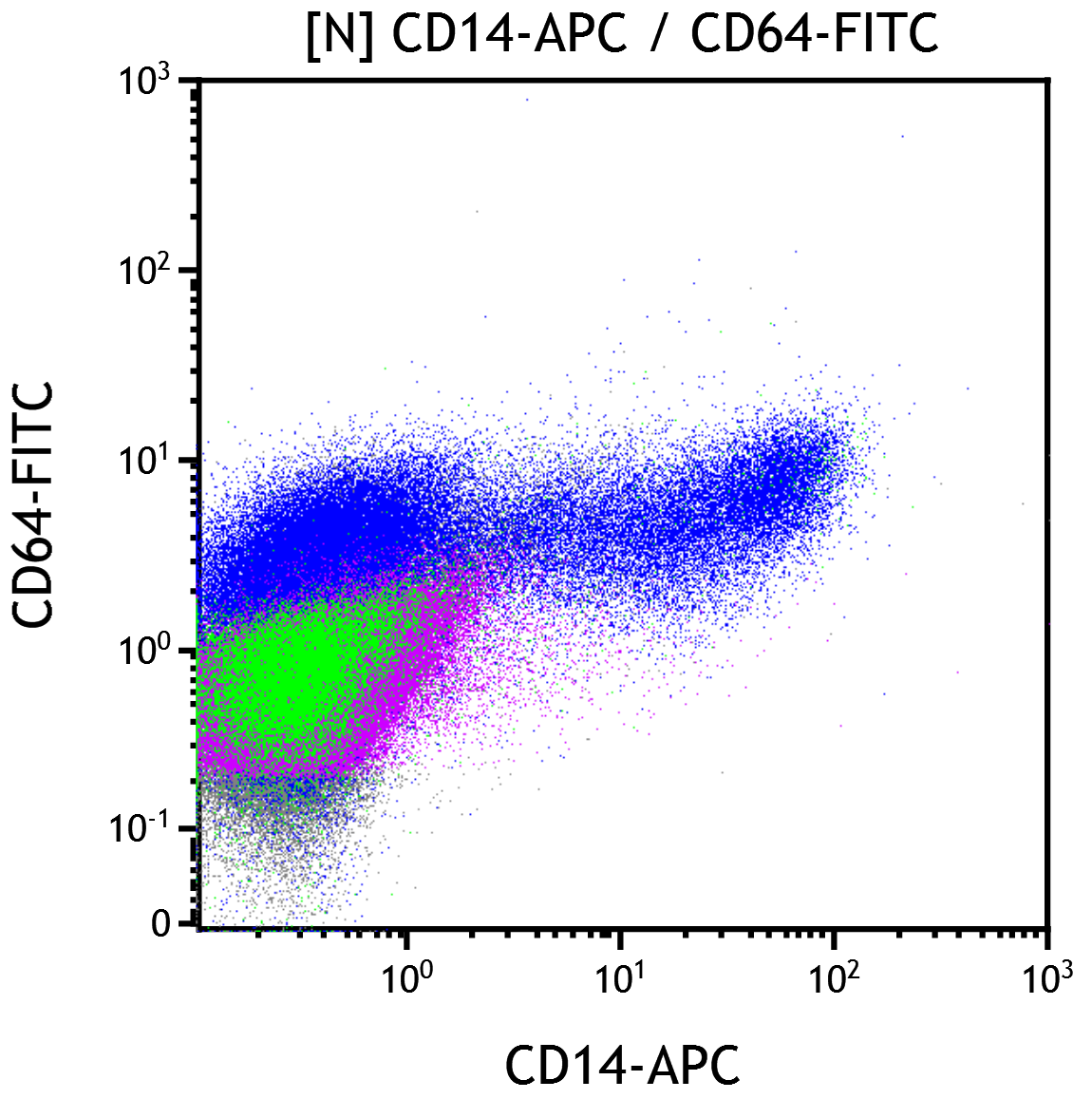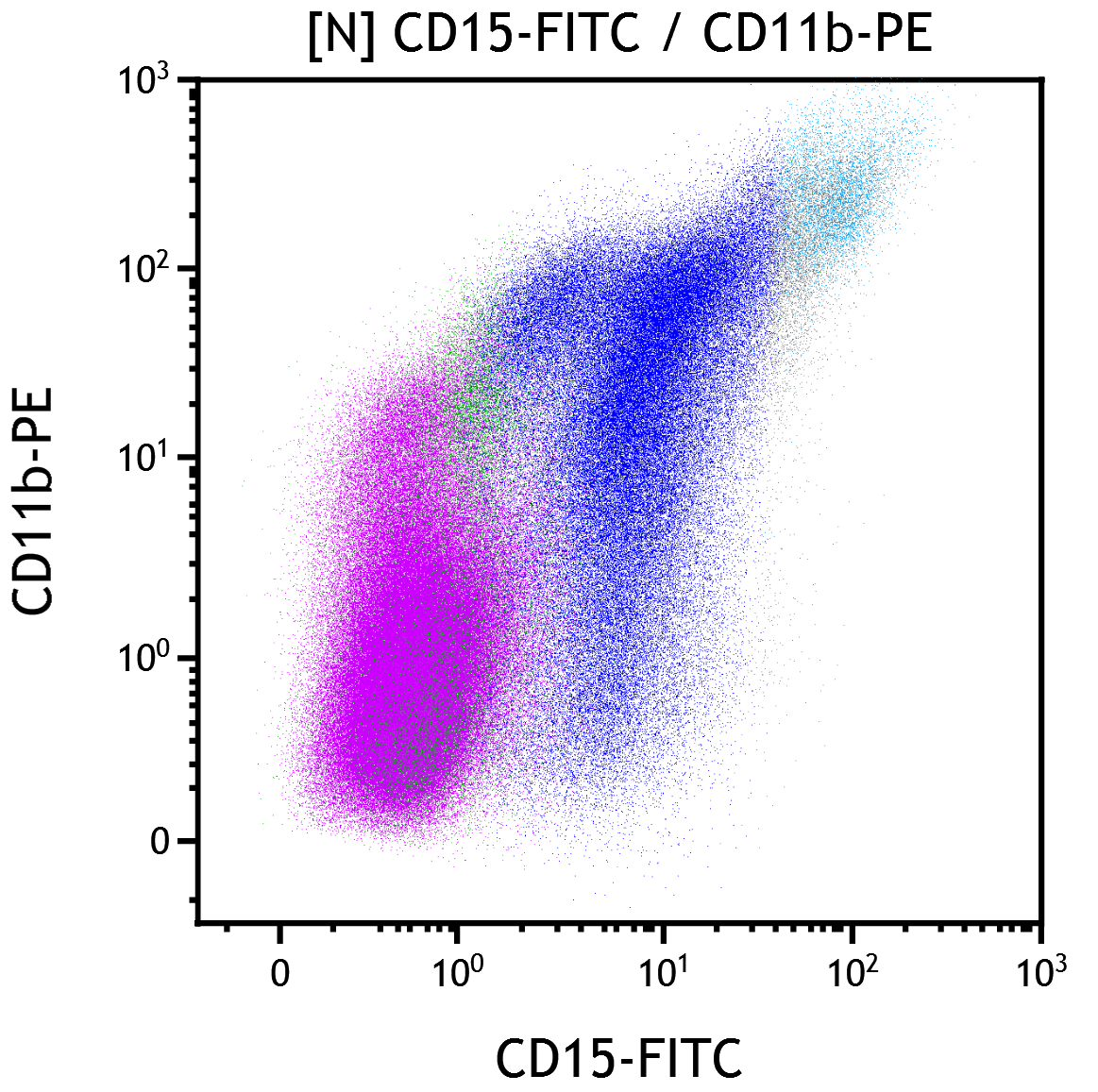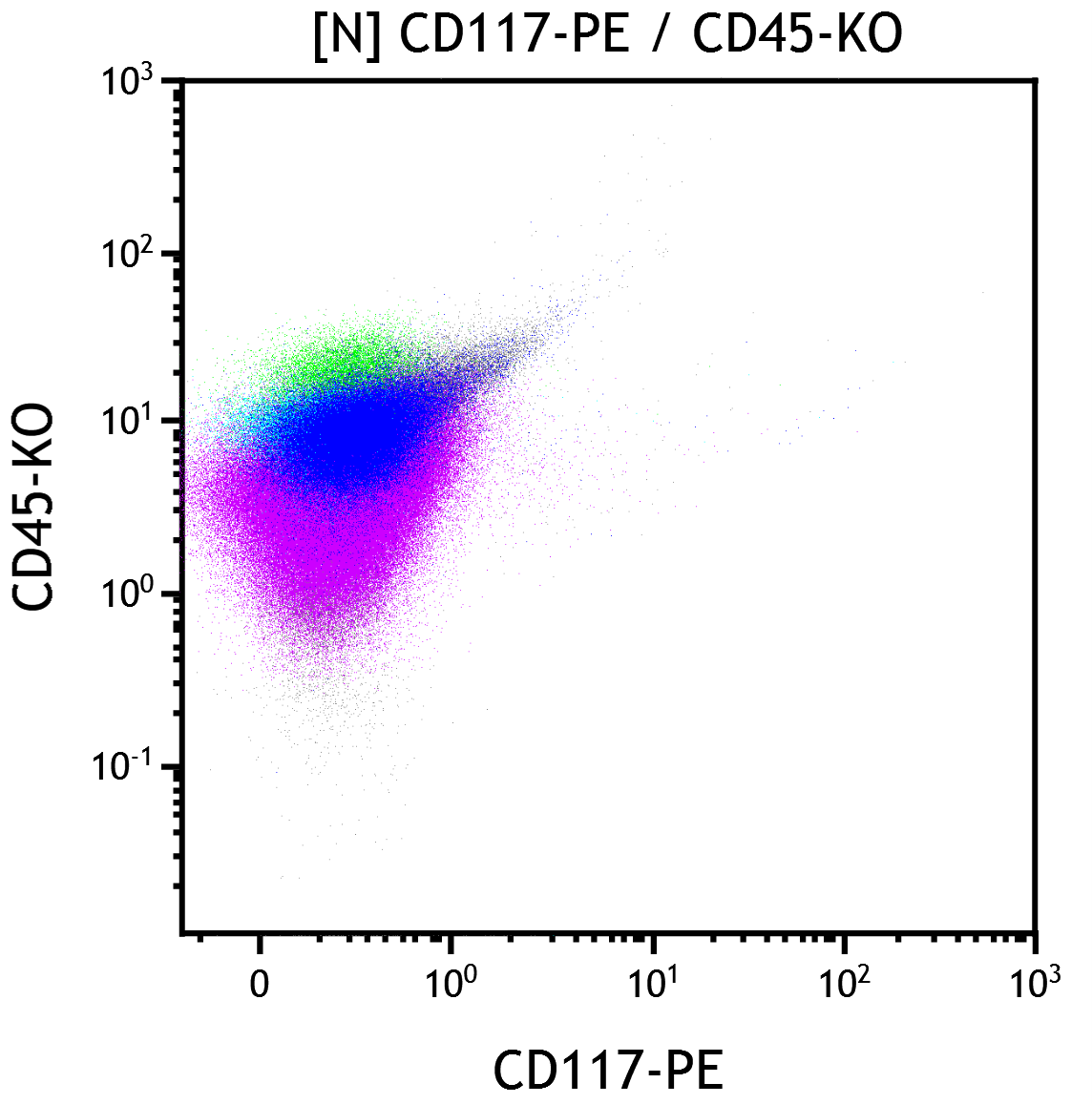**  **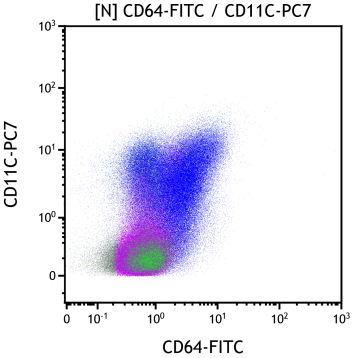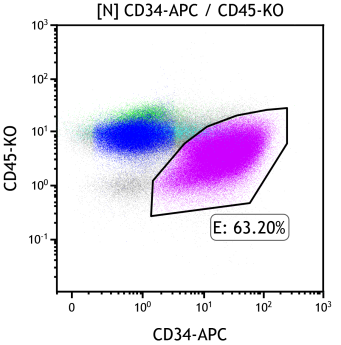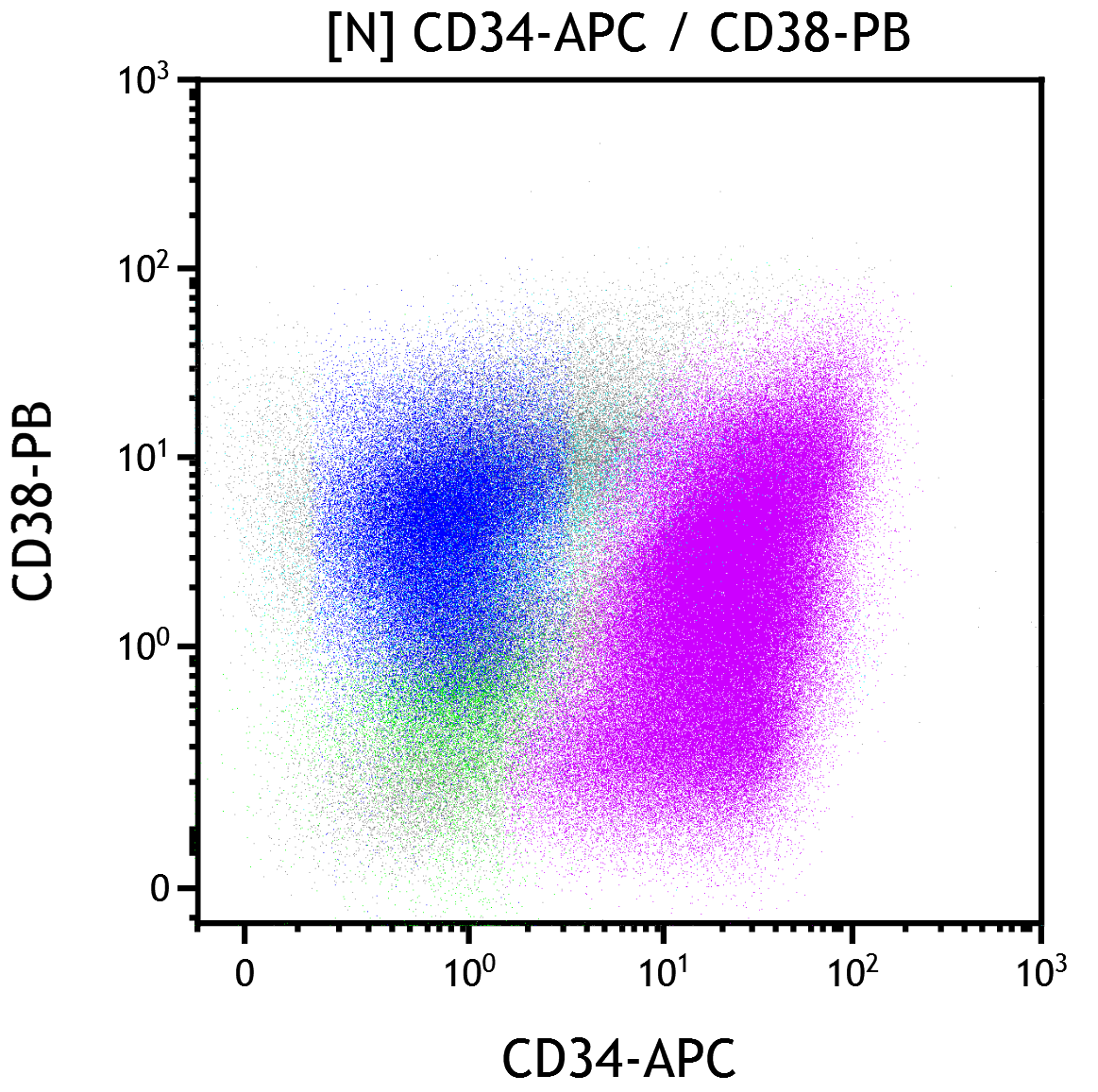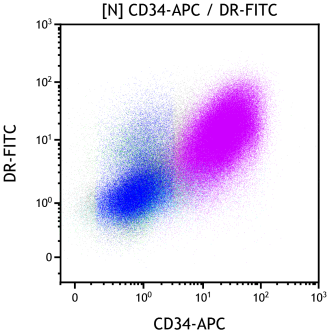** |
| --- |
| **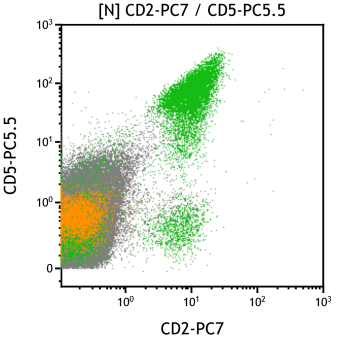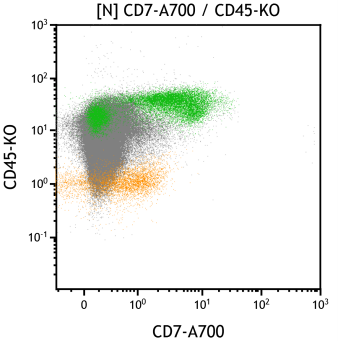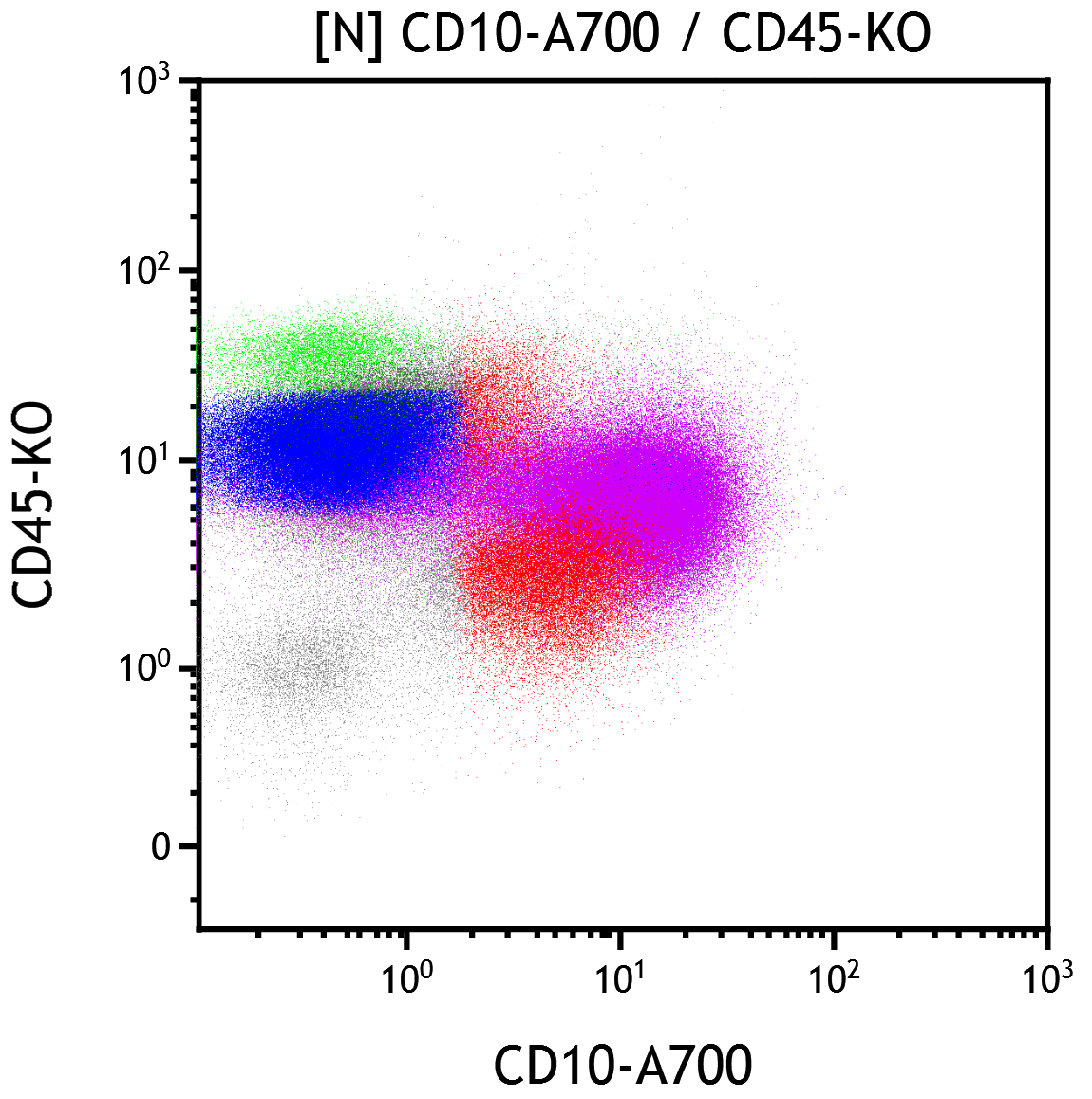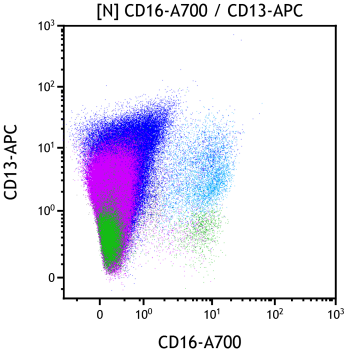**  **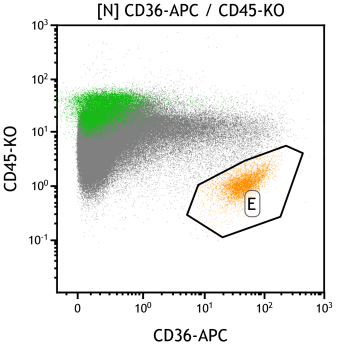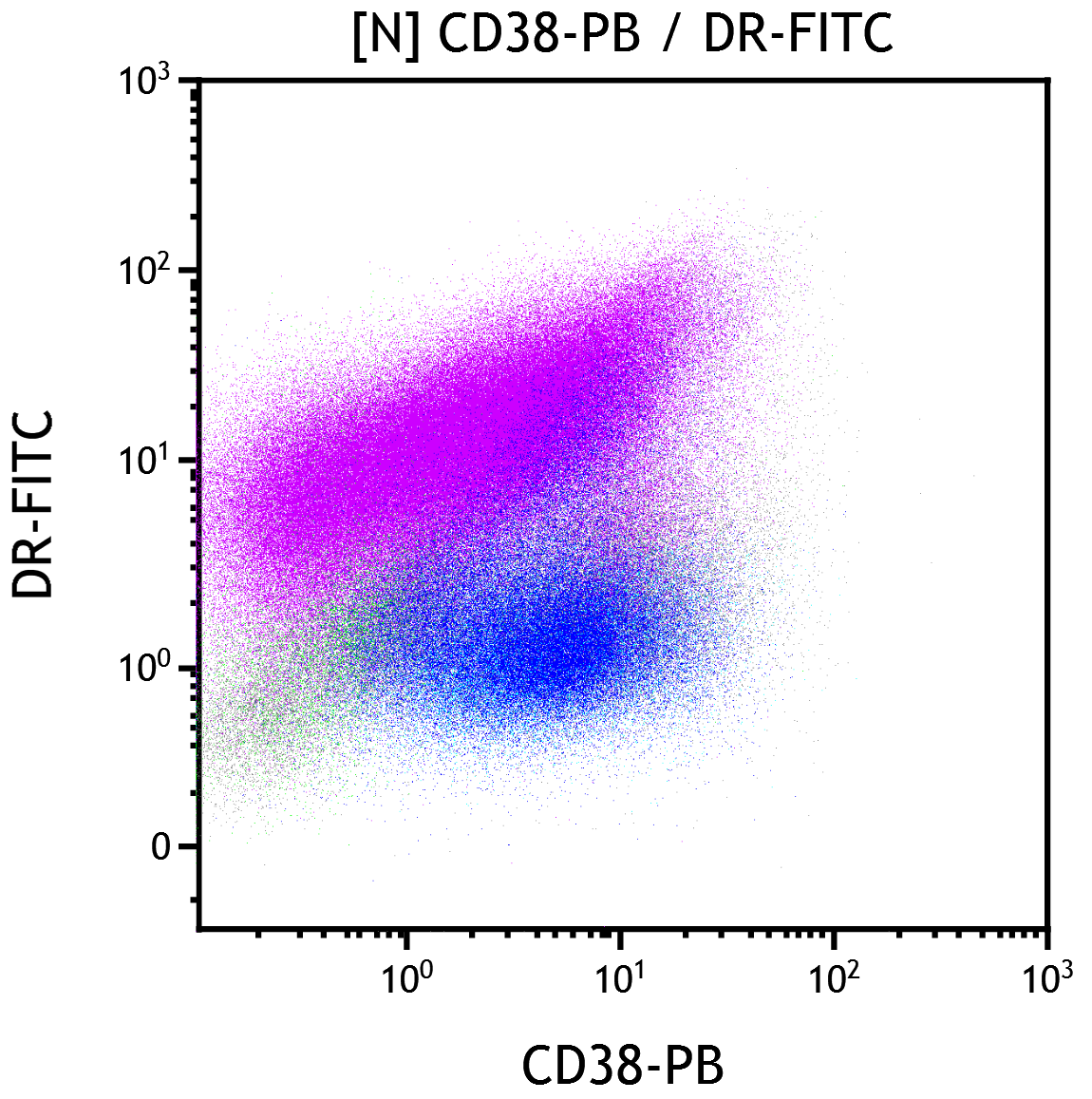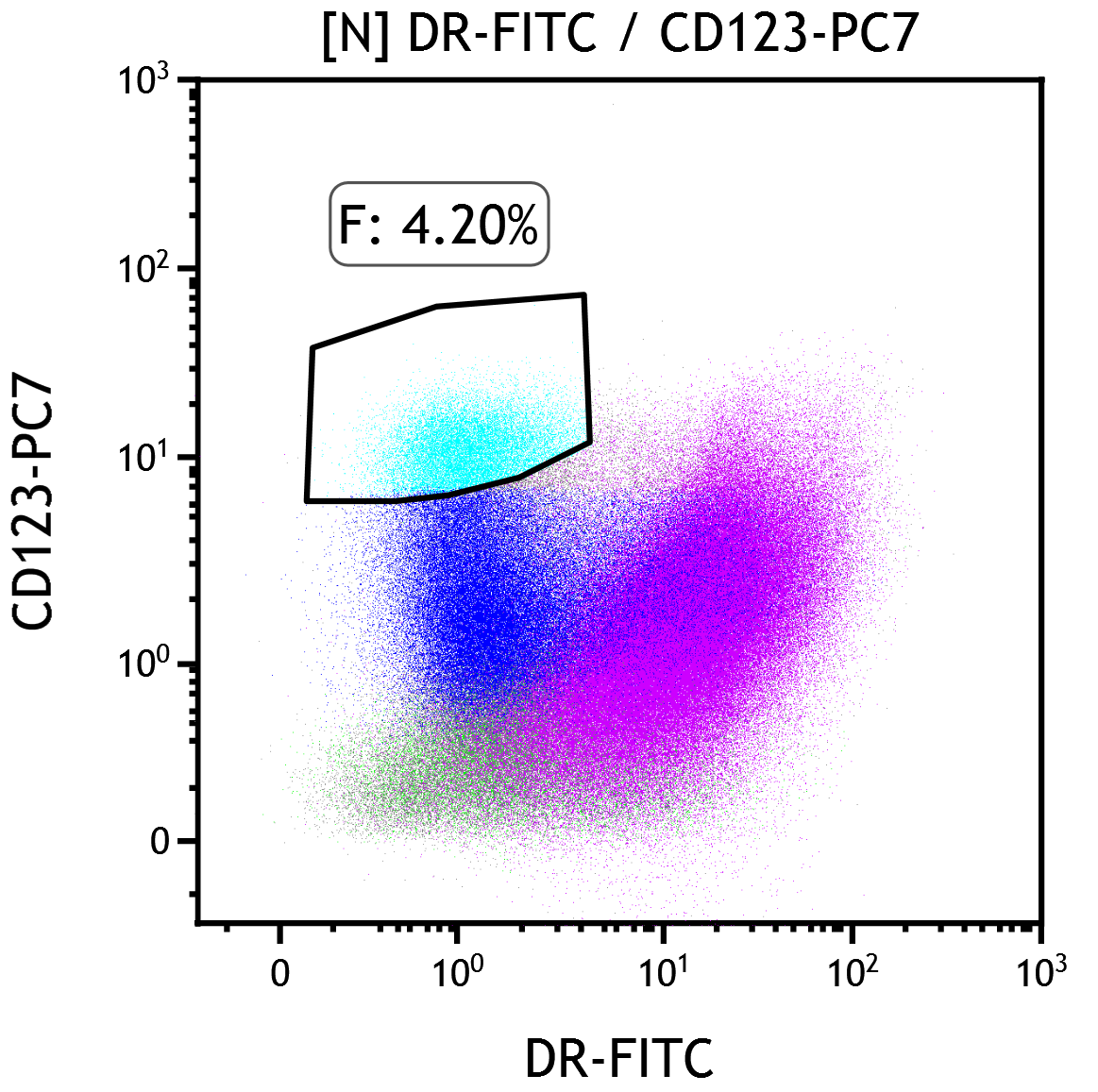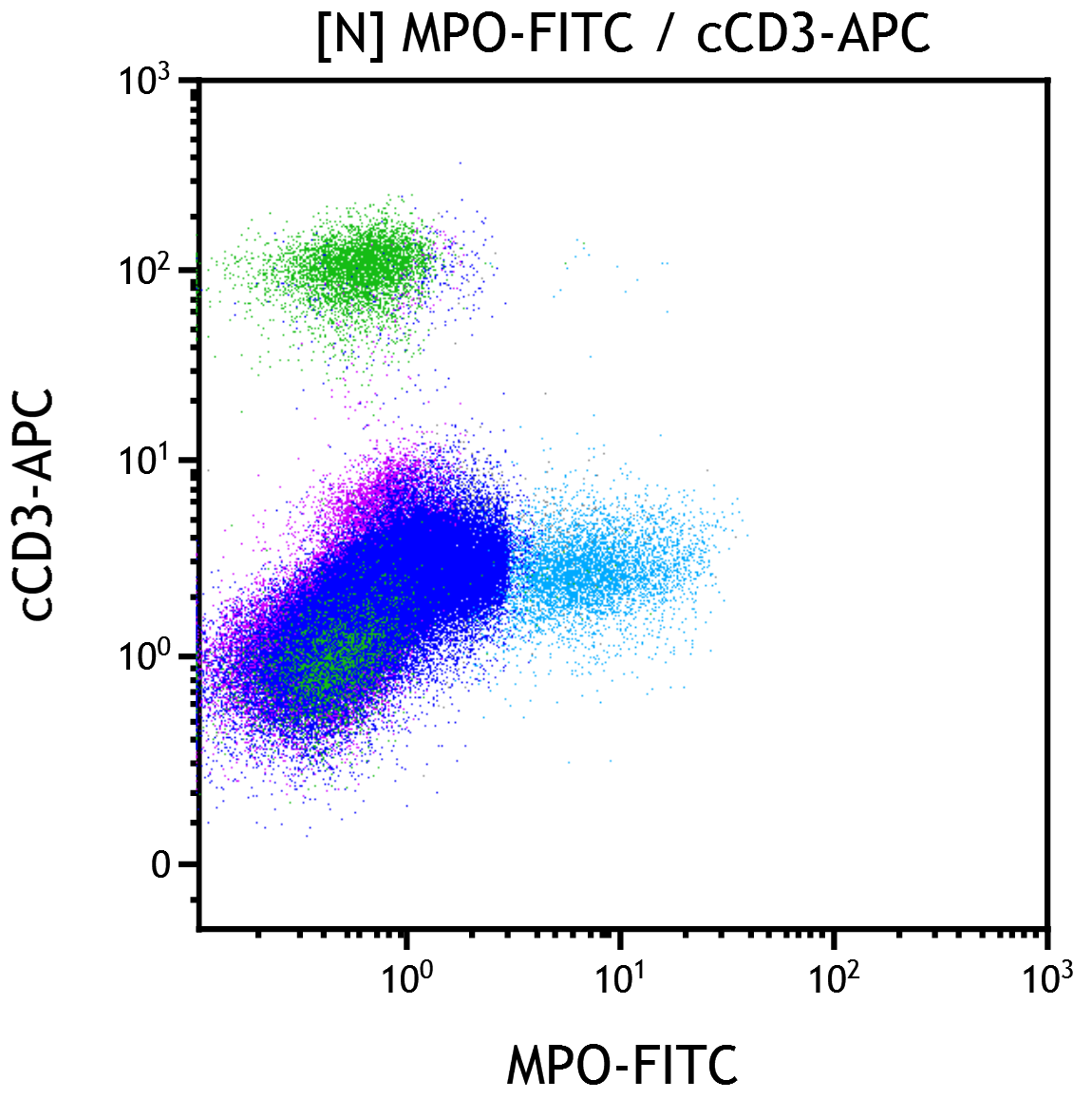** |
